# Menopause status and attitudes in a Turkish midlife female population: an epidemiological study

**DOI:** 10.1186/1472-6874-10-1

**Published:** 2010-01-11

**Authors:** Unal Ayranci, Ozgul Orsal, Ozlem Orsal, Gul Arslan, Dursun Figen Emeksiz

**Affiliations:** 1 Kurtulus Aile Sagligi Merkezi, Vatan Cd. 12/A Eskişehir, Turkey

## Abstract

**Background:**

It is a well accepted status that socio-cultural characteristics may affect the onset of menopause and its characteristics. The aims of this study were to describe the prevalence rates of menopausal symptoms and these symptoms related factors, and to assess the women's attitudes towards some climacteric issues.

**Methods:**

This survey was conducted between Jan., 1^st ^2008 and March, 31^st ^2008 to research the menopause status of the female population in a city of western Turkey. The study group consisted of 1551 women selected with a multistage area sampling method: a random sample of individuals aged 40-65 years. The questionnaire included questions pertaining to women's sociodemographic characteristics, women's menopausal status, some statements about the climacteric, use of hormones at menopause or before menopause, and some climacteric myths. The data was analyzed by Chi-square (x^2^) analysis and percent (%) ratios with a significant value of *P *< 0.05.

**Results:**

The mean age of the women was 49.1 years. Over 90% of the women were of the negative opinion that the climacteric is "the end of youth", "the end of fecundity", and "the end of unclean days". Most women said that cessation of menstrual periods was the most positive thing because they do not have to wait for monthly bleedings, use sanitary equipment, or take birth control methods. There were significant connections between age groups and nearly all the items, with the exception of the items "the end of life" and "the end of fecundity". Among the women, hot flushes were the most common complaint occurring in 96.5% of women: being severe in 32.9%, moderate in 43.1% and mild in 20.4%. This was followed by low backache or muscle pain 95.0% (25.9% severe, 46.0% moderate and mild 23.1%), headache 91.7% (21.9% severe, 34.9% moderate and 34.9% mild) and feeling tired 91.0% (15.3% severe, 38.6% moderate and 37.1% mild).

**Conclusions:**

Most of the women in this study had mixed ideas of opinions concerning the climacteric, and the majority was also suffering from climacteric complaints. This data could assist healthcare providers in the provision of culturally competent health care to midlife Turkish women.

## Background

It is well known that socio-cultural and economic factors may influence the menopausal transition; however, most reports are from developed countries, and from minority and native populations. In Western countries, menopausal symptoms such as hot flushes, sweating and vaginal dryness are considered the main climacteric complaints. In other cultures, these symptoms dramatically vary from those observed in Western women [1], with North American and European samples reporting higher rates of symptoms than Asian women [2]. Some researchers [3] also found that Japanese Americans were significantly less likely to report hot flushes during the previous 2 weeks when compared with European Americans. Japanese Americans were also significantly less likely to report experiencing other symptoms (15 of 30 in a postal sample; 6 of 30 in a monitored sample) than European Americans. It has been speculated that the reasons for these differences were possibly genetic or cultural [2,3].

In the literature on menopause, it has often been stated that it is a difficult period with many symptoms [4,5]. Besides being a major cause of morbidity such as heart disease and osteoporosis, menopause and its attendant hormonal changes also cause symptoms that affect the quality of life, such as hot flushes, night sweats, sleep disturbances, urinary frequency, vaginal dryness, poor memory, anxiety and depression [6,7]. Much of the reports has alluded to the impact of hormonal changes and decline in estrogen concentrations during menopause. Besides apparent physiological changes during the menopausal years, women in midlife often go through a social transition with increased levels of stress that may require additional coping mechanisms. Thus, the symptoms reported during mid-life may be influenced by multiple factors [5]. In a previous study, it was found that the loss of quality of life observed in middle-age women begins with the menstrual irregularities in the pre-menopause [6,7]. Epidemiological surveys on representative samples of women in menopause have shown that most problems experienced by women are also due to age [8]. One-third of women over the age of 60 complain about urogenital estrogen insufficiency symptoms. This ratio is as high as two-thirds in women over age 75 [9]. As age increases, it has been described that the risk of severe depressive symptoms increases with ovarian steroid production cessation [6]. The role of hormonal changes in the development of these symptoms, however, has continued to be discussed [10].

Menopausal symptoms can really have an important impact in the daily, social and sexual life of postmenopausal women [11]. Some population based surveys, largely conducted among Caucasian populations, have reported a high prevalence of menopausal symptoms ranging between 40% and 70% [10,12,13]. Conversely, studies on Asian women from different ethnic backgrounds have reported lower symptom prevalence rates between 10% and 50% [14]. The studies in Turkey showed a prevalence of 35%-90% [15-17]. The duration, severity and impact of these symptoms vary tremendously from person to person and population to population, but menopausal symptoms can profoundly affect personal and social functioning in affected individuals. Whether or not the psychological and emotional problems reported by some menopausal women can be attributed specifically to the menopausal transition is a controversial issue [18]. Symptoms experienced by women during and after the menopausal transition are influenced by preconceived attitudes toward the menopause, personality type, and exposure to a greater or lesser degree of life stressors [19]. There is some evidence that ethnicity and socioeconomic status may also influence the menopausal experience, either directly or indirectly, by their influence on some psychosocial factors [20].

This survey aimed to describe the prevalence rates of menopausal symptoms experienced by women in a group of midlife age group, and these symptoms related factors. Secondary objectives included determining the mean ages and associated factors of these women, as well as to assess the women's attitudes towards some items. We hypothesize that social, ethnical, economic and cultural conditions are associated with increased risks of symptom reporting.

## Methods

### Study questionnaire

The questionnaire used in this survey, based on the questions in the appendix in the article 'Women's views of the climacteric at the time of low menopausal hormone use, Estonia 1998' by Hovi et al (2004) [21], as well as literature [15,22-24], was modified to suit the Turkish culture and norms (see Additional file 1). These changes allowed the researchers to cover all the profession groups in the female general public living in the city, such as tradesmen, workers, housewives, academicians, teachers, engineers, adjudicators etc.

The questionnaire, consisting of questions in Turkish, was divided into four broad sections: the first part included women's sociodemographic characteristics; the second part included women's menopausal status at the study time and some statements about the climacteric, use of hormones at menopause or before menopause, menopause related some myths concerning the Islam religion, some opinions about menopause and attitudes and beliefs towards menopause, and this part also included women's levels of knowledge about menopause, information on taking any hormonal drug, medicine for anxiety or depression that menopause causes, drugs against sleep disturbances that menopause causes, or calcium supplements for menopause symptoms, if their social life was affected after menopause, and to what degree they cope with menopause in general. In the last part, women were asked if they had experienced symptoms in the last 6 months or since the age of 40, and to grade its severity as none, mild, moderate, and severe. This section, including symptoms experienced during menopause in women aged 40 and over, was prepared using items in the Grene Climacteric Scale [25] and also the literature [10,21]. This part also included questions concerning to what degree these complaints affected their life, if they had visited any person such as physician, psychiatrist, or nurse for those complaints, and also the respondents' source and level of information and their informational needs.

The questionnaire was then pre-tested on a sample of 74 participants from different subpopulations of the city. Alpha coefficients for the reliability and internal consistency of the questions were found to be 0.782 and 0.831 for menopause related myths originated from Islam, some opinions about menopause as well as the women's attitudes and beliefs towards menstruation and menopause, and questions concerning to what degree complaints affected their lives. The completed questionnaires were checked for consistency and completeness. Attitudes towards menopause were answered using the following options: "I agree", "Neither agree nor disagree", and "I disagree". Women were asked to fill in the section regarding "level of complaints experienced" as percentages ranging between severe and none. Responses to all the items in this section were converted to a score between 1 and 3. The information obtained on symptom reporting was based on the two weeks prior to the study rather than a more distant past, thus helping minimize problems with recall.

### Sampling

1551 women were interviewed face-to-face between January and March 2008 to determine on the menopause status of the female population in a city of western Turkey, and the questionnaires were presented to them during the interview session. Due to the length of the questionnaire, the survey being conducted on a general population, and there being a possibility that some people in the city are illiterate, the researchers helped to explain any questions that the respondents found incomprehensible. 1727 houses situated in the 71 quarters of the city, each having approximately equal populations, were determined using a stratified random sample method. During the study period, a total of 1986 women aged 40 and over were living in these places. The study was conducted at the participants' houses. Our objective was to contact the whole population of subjects in the aforementioned houses. Criteria for inclusion in the study were having the ability to understand the questionnaire either by themselves or with the help of the researchers, and being aged 40 and over.

Those who wanted to interview with the researchers together with her husband, friend or daughter (n = 39), who had any mentally disability (n = 22), or who had any genital malformations or disease including malignancy and premature ovarian failure affecting menstruation or menopause (n = 3) were excluded from the study. In addition, those not willing to participate without indicating any reason (n = 103), and those not being at home at the time of the study (n = 168) were also excluded. In addition, no women aged 70 and over were included among those agreeing to participate in the study. The remaining sample (n = 1551) was representative. It did not differ from the female general population aged 40 and over in terms of age.

### Procedures

Data was obtained by visiting the houses of women in the city of Eskisehir. All subjects (1551/1986, 78.1%) were told that participation in the investigation was strictly voluntary and that the data collected would not be used for anything except the research aim. Those who agreed to participate were given the questionnaire to complete. The interval for completing the questionnaire was approximately half an hour per subject. The investigators met weekly to ensure the quality of data collected.

Following the completion of the questionnaires and inventories, their body mass indexes (BMI) were calculated by measuring their heights and weights. Women were considered to be obese or overweight if they had a Body Mass Index (BMI) of 25 kg/m^2 ^or more [26]. Each women's body weight was measured with domestic scales and height with a meter rule, and their BMI was calculated using the formula [BMI = weight (kg)/(height^2 ^(m)].

### Menopausal definitions

We adapted the menopausal status groups' classification as defined by the World Health Organization [27]. Premenopausal women had experienced only regular menstrual bleeding within the last 12 months. Perimenopausal women were defined as those women who had experienced irregular menses within the last 12 months or an absence of menstrual bleeding for more than 3 months but less than 12 months. Moreover, to aid statistical analysis, two additional menopausal status groups were defined in our study population. Postmenopausal-natural women had stopped menstrual bleeding spontaneously at least a year previously. Postmenopause-surgical women had stopped having periods as a result of medical (e.g. chemotherapy or radiotherapy to the ovaries) or surgical intervention (e.g. hysterectomy or oopherectomy or both).

### Data analysis

The data were analyzed by using the computer software package Statistical Package for Social Sciences (SPSS, Chicago, Il, USA) for Windows version 11.5 (13.0.1). The statistical analysis was carried out using the Student's t test for continuous variables and Chi-square (x^2^) test for categorical variables. The prevalence of each menopausal symptom was calculated. Results are given as numbers and percentages (%) with 95% Confidence Interval (CI). A value of p < 0.05 was considered statistically significant unless otherwise specified.

### Ethical approval

The permission for the study was obtained by making a petition prior to collecting data. This was achieved by contacting and receiving approval from the Director of the Institution of Eskisehir Osmangazi University. Participants completed an informed consent form in which they were assured of the confidentiality of their responses following which they provided informed verbal consent that participation was voluntary and anonymous. It was also stated that the participants' responses were unidentifiable. All women gave their informed consent prior to their inclusion in the study.

## Results

There were significant differences in all the socioeconomic characteristics and self-rated health between all the age-groups. Most women were in the age group 40-44 (28.4%), followed by the age groups 45-49 (25.3%) and 50-54 (25.5%). Nearly three quarters of the women were overweight and obese (74.7%), which was found to be the highest in the age groups 55-59 and 65-69 (86.1% and 78.4%, respectively). Most women reported that they were married (86.8%). Education levels were low: only 550 women (35.4%) had completed high school, university or an institute. At the time of recruitment, 460 women (29.7%) were employed outside home full- or part-time, and 1091 (70.3%) were unemployed. Women in the older age-groups were less often employed outside the home, being the highest in age group 50-54 (34.7%). All the women listed their primary nationality/religion as Turkish and Islam (Unshown datum in Table). 537 (34.6%) and 48 (3.1%) women reported smoking cigarette and consuming alcohol, respectively. In the older age-groups women reported that they smoked fewer cigarettes; while this proportion ranged between 31.1% and 47.6% in those aged between 40 and 54, it ranged between only 11.5% and 17.7% in those aged between 55 and 69. In terms of alcohol consumption, the women reported that they consumed less alcohol as their age increased; namely in those aged 40-44 this proportion was 5.4%, while it was lower (between 0.0% and 3.9%) in the other age groups, with no proportion reported in the age group 65-69.

The proportion of women who reported doing either regular or irregular exercise was only 3.3%, with this figure reported at 0.0% in the older age groups (60-64 and 65-69). Almost all of the women interviewed (96.9%) were one or more parous. 64 women had only 1 child, 463 women had 2 children and the 976 remaining women had 3 and over children (62.9%). Most of the women reported family income as average and high (72.1%). Fourty five percent of the women rated their health as very good/good, and 46.4% as average. Most women had at least one of the chronic diseases (83.2%), which was more frequent in older age groups. Of these, the most common long-term health problems diagnosed by a physician were rheumatic arthritis (26.7%), diabetes mellitus (11.4%), and hypertension (11.3%), followed by muscle and joint problems (7.2%) and anemia (6.7%). The respondents' detailed demographic characteristics, and self-rated health status by their age groups are presented in Table 1.

**Table 1:** The respondents' demographic characteristics, and self-rated health

	40-44 n(%) 441(28.4)	45-49 n(%) 393(25.3)	50-54 n(%) 395(25.5)	55-59 n(%) 209(13.5)	60-64 n(%) 51(3.3)	65-69 n(%) 62(4.0)	All n(%) 1551(100)
*Body Mass Index*	x^2^= 107.8; DF = 10; p = 0.000						
≤ 24.99	116(26.3)	136(34.6)	86(21.8)	29(13.9)	12(23.5)	12(21.0)	392(25.3)
≥ 25.00 - ≥ 29.99	239(54.2)	157(39.9)	181(45.8)	73(34.9)	16(31.4)	19(30.0)	683(44.2)
≥ 30	86(19.5)	100(25.4)	128(32.4)	107(51.2)	23(45.1)	30(48.4)	474(30.5)
*Marriage status*	x^2^=26.3; DF = 5; p = 0.000						
Single (widow/separated)	377(85.5)	335(85.2)	355(89.9)	194(92.8)	368(70.6)	49(79.0)	1346(86.8)
Married	64(14.5)	58(14.8)	40(10.1)	15(7.2)	15(29.4)	13(21.0)	205(13.2)
*Education level*	x^2^= 130.1; DF = 15; p = 0.000						
Illiterate	14(3.2)	5(1.3)	12(3.0)	13(6.2)	6(11.8)	14(22.6)	64(4.1)
Primary education (primary and secondary school)	268(60.8)	203(51.7)	246(62.3)	145(69.4)	32(62.7)	43(69.4)	937(60.4)
High school	85(19.3)	84(21.4)	45(11.4)	26(12.4)	4(7.8)	0(0.0)	244(15.7)
University or institute	74(16.8)	101(25.7)	92(23.3)	25(12.0)	9(17.6)	5(8.1)	306(19.7)
*Employment status*	x^2^= 17.5; DF = 5; p = 0.004						
Housewife	319(72.3)	267(67.9)	258(65.3)	153(73.2)	40(78.4)	54(87.1)	1091(70.3)
Employed outside home	122(27.7)	126(32.1)	137(34.7)	56(26.8)	11(21.6)	8(12.9)	460(29.7)
*Smoking*	x^2^= 120.9; DF = 5; p = 0.000						
Smoker	210(47.6)	166(42.2)	123(31.1)	24(11.5)	3(5.9)	11(17.7)	537(34.6)
Non-smoker	231(52.4)	227(57.8)	272(68.9)	185(88.5)	48(94.1)	51(82.3)	1014(65.4)
*Alcohol consumption*	x^2^= 14.3; DF = 5; p = 0.014						
Yes	24(5.4)	6(1.5)	9(2.3)	7(3.3)	2(3.9)	0(0.0)	48(3.1)
No	417(94.6)	387(98.5)	386(97.7)	202(96.7)	49(96.1)	62(100.0)	1503
*Participation in sport/exercise*	x^2^= 48.5; DF = 10; p = 0.000						
Regular	28(6.3)	10(2.5)	10(2.5)	3(1.4)	0(0.0)	0(0.0)	51(3.3)
Irregular	299(67.8)	288(73.3)	321(81.3)	174(83.3)	36(70.6)	40(64.5)	1158(74.7)
No (including former)	114(25.9)	95(24.2)	64(16.2)	32(15.3)	15(29.4)	22(35.5)	342(22.1)
*Parity*	x^2^= 18.4; DF = 5; p = 0.002						
Nulliparous	22(5.0)	17(4.3)	9(2.3)	0(0.0)	0(0.0)	0(0.0)	48(3.1)
Parous	419(95.0)	376(95.7)	386(97.7)	209(100.0)	51(100.0)	62(100.0)	1503(96.9)
*Family income*	x^2^= 49.4; DF = 10; p = 0.000						
Low	125(28.3)	105(26.7)	115(29.1)	42(20.1)	20(39.2)	26(41.9)	433(27.9)
Average	233(52.8)	216(55.0)	164(41.5)	127(60.8)	26(51.0)	32(51.6)	798(51.5)
High	83(18.8)	72(18.3)	116(29.4)	40(19.1)	5(9.8)	4(6.5)	320(20.6)
*Self-rated health*	x^2^= 340.1; DF = 10; p = 0.000						
Very good or good	292(66.2)	185(47.1)	127(32.2)	52(24.9)	18(35.3)	24(38.7)	698(45.0)
Average	145(32.9)	200(50.9)	230(58.2)	83(39.7)	29(56.9)	33(53.2)	720(46.4)
Poor or very poor	4(0.9)	8(2.0)	38(9.6)	74(35.4)	4(7.8)	5(8.1)	133(8.6)
*Any chronic disease*	x^2^= 40.5; DF = 5; p = 0.000						
Yes	330(74.8)	324(82.4)	344(87.1)	190(90.9)	44(86.3)	58(93.5)	1290(83.2)
No	111(25.2)	69(17.6)	51(12.9)	19(9.1)	7(13.7)	4(6.5)	261(16.8)

The mean age of all the women was 49.1 ± 6.4 (40-69, n = 1551). This figure was 41.5 ± 1.1 (40-44, n = 271) for the premenopausal women, 44.8 ± 2.5 (40-48, n = 275) for the perimenopausal women, 52.9 ± 5.1 (42-69, n = 823) for the postmenopausal-natural women, and 50.2 ± 5.8 (41-65, n = 182) for the postmenopausal-surgical women. Consistent with the age distribution of the sample, more than half of the women had experienced natural menopause (53.1%) and 182 (11.7%) had surgical menopause (Unshown data in Table 1).

Table 2 presents the respondents' opinions, attitudes and misconceptions towards some statements about the climacteric and hormones used during manopause and postmenopause. One hundred percent of the women gave their responses on the statements concerning the climacteric; disagree, neither agree nor disagree, or agree. Over 90% of the women were of the negative opinion that the climacteric is "the end of youth", "end of fecundity", and "end of impurity days". Interestingly, about one third of the women (32.0%) believed that "divorce rate could increase in this period", and also that the climacteric is a "period for cuckolding", 21.3%. Mostly, women said that cessation of the menstrual periods was the most positive thing because they do not have to wait for irregular bleeding, use sanitary equipment, take birth control methods, and they are not at risk of getting pregnant. In addition, most women stated that they can start a new period in their lives, and they feel calm, independent and mature (89.4%). Thirty two percent of the women agreed with the opinion that "menopausal women do not need treatment by a physician", 29.0% with the opinion "hormones should be given to all menopausal women", and 89.0% with the opinion "hormones should be given to all women experiencing menopausal symptoms."

**Table 2:** The respondents' attitudes towards some statements about climacteric and hormones used during climacteric and postmenopause,

The respondents' attitudes and misconceptions towards menopause (Alpha coefficient: 0.782)	Disagree N(%)	Neither agree nor disagree n(%)	Agree n(%)
*Negative features*			
It is the end of youth	92(5.9)	53(3.4)	1406(90.7)
It is the end of life	1498(96.6)	16(1.0)	37(2.4)
It is the beginning of getting older	112(7.2)	109(7.0)	1330(85.8)
It is the end of fecundity	28(1.8)	9(0.6)	1514(97.6)
The divorce rate could increase in this period	744(48.0)	310(20.0)	497(32.0)
I cannot endure problems from child or spouse in this period	797(51.4)	280(18.1)	474(30.6)
It is the end of uncleanness days	34(2.2)	22(1.4)	1495(96.4)
It is the beginning of distress	966(62.3)	158(10.2)	427(27.5)
It is a period for cuckolding	981(63.2)	239(15.4)	331(21.3)
*Positive attitudes*			
I will not be obliged to use sanitary pads	78(5.0)	50(3.2)	1423(91.7)
I will experience no distress such as irregular menses	88(5.7)	84(5.4)	1379(88.9)
I will not be in need of using birth control methods	57(3.7)	53(3.4)	1441(92.9)
I will be at no risk for getting pregnant	82(5.3)	141(9.1)	1328(85.6)
Woman feels herself to be in the prime of life	61(3.9)	103(6.6)	1387(89.4)
It is a period that must be experienced	155(10.0)	56(3.6)	1340(86.4)
*Opinions*			
It is not necessary to consult a doctor for treatment in this period	795(51.3)	260(16.8)	496(32.0)
Hormones should be given to all menopausal women	895(57.7)	206(13.3)	450(29.0)
Hormones should be given to all women having menopausal symptoms	114(7.4)	56(3.6)	1381(89.0)
*Misconceptions*			
It is a punishment that God gave to women	1074(69.2)	128(8.3)	349(22.5)
It is a reward that God gave to women for stopping dirtiness days	1074(69.2)	128(8.3)	349(22.5)

Table 3 shows the relationship between the respondents' age groups and their attitudes, all answered at the highest rates, towards some statements about climacteric and hormones used during the climacteric and postmenopause. There were significant connections between age groups and nearly all the items, with the exception of the items "end of life" and "end of fecundity". All of the women reporting "agree" or "disagree" were in the age groups 50-54 and less. In general, the proportions of those reporting "agree" and "disagree" showed a decrease with an increase in the age of the responder, while there were little changes in some age groups. Most of those agreeing with the items (End of youth, Beginning of getting older, End of fecundity, End of unclean days, I will not be obliged to use sanitary pads, I will experience no distress such as irregular menses, I will not be in need of birth control methods, A period that must be experienced, Hormones should be given to all menopausal women, and Hormones should be given to all women having menopausal symptoms) were in the age group 40-44, the youngest age group, whereas most of those agreeing with the items 'I will be at no risk of getting pregnant', and 'Woman feels herself to be in the prime of life' were in the age group 50-54. The most frequently encountered age group for those disagreeing with the items 'the end of life', 'It is not necessary to consult a doctor for treatment in this period', 'It is the punishment that God gave women' and 'The end of unclean days is a reward that God gave to women' were 40-44, and also 50-54 for the items 'the divorce rate could increase in this period', 'I cannot endure problems from my child or spouse in this period', 'the beginning of distress' and 'the period for cuckolding'.

**Table 3:** The relationship between the respondents' age groups and theirattitudes towards some statements about climacteric and hormones used during climacteric and postmenopause (Agree and disagree were preferenced by the highest answer rates)

	40-44 n(%) 441(28.4)	45-49 n(%) 393(25.3)	50-54 n(%) 395(25.5)	55-59 n(%) 209(13.5)	60-64 n(%) 51(3.3)	65-69 n(%) 62(4.0)	All n(%) 1551(100.0)
End of being youth*							
Agree	395(28.1)	356(25.3)	361(25.7)	195(13.9)	49(3.5)	50(3.6)	1406(90.7)
End of life							
Disagree	424(28.3)	378(25.2)	383(25.6)	206(13.8)	49(3.3)	58(3.9)	1498(96.6)
Beginning of getting older*							
Agree	373(28.0)	314(23.6)	355(26.7)	185(13.9)	49(3.7)	54(4.1)	1330
End of fecundity							
Agree	427(28.2)	382(25.2)	394(26.0)	200(13.2)	51(3.4)	60(4.0)	1514(97.6)
Divorce rate could increase in this period**							
Disagree	173(23.3)	153(20.6)	235(31.6)	138(18.5)	15(2.0)	30(4.0)	744(48.0)
I can not endure problems from child or spouse in this period**							
Disagree	209(26.2)	170(21.3)	235(29.5)	131(16.4)	18(2.3)	34(4.3)	797(51.4)
End of impurity days*							
Agree	418(28.0)	379(25.4)	387(25.9)	204(13.6)	51(3.4)	56(3.7)	1495(96.4)
Beginning of distress**							
Disagree	210(21.7)	250(25.9)	278(28.8)	168(17.4)	28(2.9)	32(3.3)	966(62.3)
Period for cuckolding**							
Disagree	214(21.8)	241(24.6)	301(30.7)	164(16.7)	30(3.1)	31(3.2)	981(63.2)
I will not be obliged to use sanitary pad**							
Agree	387(27.2)	353(24.8)	369(25.9)	207(14.5)	49(3.4)	58(4.1)	1423(91.7)
I will experince no distress such as irregular menses**							
Agree	379(27.5)	346(25.1)	357(25.9)	198(14.4)	48(3.5)	51(3.7)	1379(88.9)
I will not be in need of using birth control methods*							
Agree	401(27.8)	349(24.2)	377(26.2)	206(14.3)	49(3.4)	59(4.1)	1441
I will be at no risk for getting pregnant**							
Agree	357(26.9)	320(24.1)	362(27.3)	196(14.8)	43(3.2)	50(3.8)	1328(85.6)
Woman fells herself in the prime of life**							
Agree	363(26.2)	343(24.7)	375(27.0)	200(14.4)	49(3.5)	57(4.1)	1387(89.9)
A period that must be experienced**							
Agree	398(29.7)	317(23.7)	345(25.7)	186(13.9)	39(2.9)	55(4.1)	1340(86.4)
It is not necessary a doctor's treatment in this period**							
Disagree	249(31.3)	183(23.0)	200(25.2)	109(13.7)	24(13.7)	30(3.0)	795(51.3)
Hormones should be given to all menopausal women**							
Agree	265(29.6)	203(22.7)	236(26.4)	126(14.1)	26(2.9)	39(4.4)	895(57.7)
Hormones should be given to all women having menopausal symptoms**							
Agree	387(28.0)	325(23.5)	371(26.9)	194(14.0)	50(3.6)	54(3.9)	1381(89.0)
It is the punishment that God gave women**							
Disagree	332(30.9)	259(24.1)	251(23.4)	145(13.5)	35(3.3)	52(4.8)	1074(69.2)
The end of unclean days is a reward that God gave to women**							
Disagree	332(30.9)	259(24.1)	251(23.4)	145(13.5)	35(3.3)	52(4.8)	1074(69.2)

Those reporting less attitudes and neither agree nor disagree are not shown in Table.

*p < 0.05; **p < 0.001

When we take a look at the relationship between the respondents' educational levels and their attitudes towards some statements about the climacteric and hormones used during the climacteric and postmenopause, there were significant relationships between age groups and nearly all the items, with the exception of only one item: "the end of life" (p >0.05). In all of the items, most of the women reporting "agree" or "disagree" were in the group primary education (60.4%), followed by the group university education (19.7%). Most of those agreeing to the items 'the end of youth', 'the beginning of getting older', 'the end of fecundity', 'the end of unclean days', 'I will not be obliged to use sanitary pads', 'I will experience no distress such as irregular menses' and 'Hormones should be given to all menopausal women' were in the education group primary, as well as most of those disagreeing with the items ' the end of life', 'the divorce rate could increase in this period', '.I cannot endure problems from child or spouse in this period', 'the beginning of distress' and 'the period for cuckolding'. Nearly one third of women agreeing with the items 'I will not be in need of birth control methods', 'A period that must be experienced' and 'Hormones should be given to all women having menopausal symptoms' were in the education group illiterate (27.8%, 28.7% and 28.0%, respectively). For the items 'It is not necessary to consult a doctor for treatment in this period', 'It is the punishment that God gave women' and 'The end of unclean days is a reward that God gave to women', most women disagreeing to those items were in the education group illiterate (31.3%, 30.9% and 30.9%, respectively), while most of women agreeing to the items 'I will be at no risk of getting pregnant' and 'Woman feels herself to be in the prime of life' were in the education group high school (26.9% and 27.0%, respectively) (Unshown data in Table).

Over one third of the women had received hormone replacement therapy (HRT) prior to the time of the study (n = 550, 35.4%. The menstrual status for a large proportion of the women receiving HRT (79.3%) was peri-, post-natural and post-surgical menopause, with the highest rate seen in those who were post menopause-natural (44.4%), followed by perimenopause (23.8%), premenopause (20.7%) and postmenopause-surgical (11.1%) (p < 0.001). A small proportion of the women (n = 48, 3.1%) reported that they have used a medicine for the distress that menopause causes, and 13.0% of the women (n = 201) reported that they had used in the past and stopped. Only 71 women indicated that they have used a medicine for the insomnia that menopause causes (n = 71, 4.6%). The proportion of those who have taken calcium supplements associated to menopause or preventing menopause was 14.1% (n = 218), with the proportion of those having used in the past and stopped 38.0% (n = 590). Half of those taking calcium supplement were postmenopausal-natural women (110/218, 50.0%), followed by premenopause (53/218, 23.9%), perimenopause (41/218, 20.2%) and postmenopause-surgical (14/218, 6.0%) (Unshown data in Table).

The prevalence of climacteric complaints by age groups is presented in Table 4. The frequencies of the climacteric complaints showed important differences according to age groups, with the exception of only the symptom "an inclination to a fracture in any bone". "sleep disturbances, 89.5%", "loss of appetite, 50.8%", "persistent cough, 54.9%", "sore throat, 56.5", and "shortness of breath, 47.8%" were the highest in the age group 40-44 in comparison with the other age groups, whereas "vaginal dryness, 60.1%", "avoiding intimacy, 48.9%", and "decrease in sexual desire, 48.9%" were the highest in the age group 45-49 when compared to the other age groups.

**Table 4:** The incidence of climacteric complaints by age groups

Symptoms	40-44 n(%) 441(28.4)	45-49 n(%) 393(25.3)	50-54 n(%) 395(25.5)	55-59 n(%) 209(13.5)	60-64 n(%) 51(3.3)	65-69 n(%) 62(4.0)	All n(%) 1551(100.0)	Statistical analysis x^2^; DF; p
Hot flushes	428(97.1)	367(93.4)	383(97.0)	207(99.0)	51(100.0)	61(98.4)	1497(96.5)	18.5; 5; 0.002
Night sweats	406(92.1)	319(81.2)	354(89.6)	195(93.3)	43(84.3)	56(90.3)	1373(88.5)	32.6; 5; 0.000
Excessive sweats	361(81.9)	273(69.5)	331(83.8)	171(81.8)	31(60.8)	49(70.9)	1216(78.4)	39.2; 5; 0.000
Sleep disturbances	395(89.5)	299(76.1)	351(88.9)	179(13.7)	32(85.6)	52(83.9)	1308(84.3)	53.8; 5; 0.000
Pins and needles in arms and legs	383(86.8)	297(75.6)	344(87.1)	185(88.5)	32(62.7)	45(72.6)	1286(82.9)	48.6; 5; 0.000
Palpitations	320(72.6)	259(65.9)	304(77.0)	163(78.9)	32(62.7)	41(66.1)	1119(72.1)	19.1; 5; 0.002
Loss of appetite	224(50.8)	196(49.9)	174(44.1)	65(31.1)	21(41.2)	29(46.8)	709(45.7)	26.1; 5; 0.000
Dizzy spells	313(71.0)	309(78.6)	287(72.7)	169(80.9)	41(80.4)	43(69.4)	1162(74.9)	13.4; 5; 0.020
Vaginal dryness	254(57.6)	236(60.1)	210(53.2)	75(35.9)	27(52.9)	37(59.7)	839(54.1)	36.6; 5; 0.000
Burning while urinating	281(63.7)	292(74.3)	309(78.2)	169(80.9)	43(84.3)	40(64.5)	1134(73.1)	37.3; 5; 0.000
Urinating more frequently	290(65.8)	284(72.3)	315(79.7)	171(81.8)	35(68.6)	42(67.7)	1137(73.3)	30.7; 5; 0.000
Urinary tract infection	274(62.1)	273(69.5)	288(72.9)	171(81.8)	44(86.3)	40(64.5)	1090(70.3)	36.0; 5; 0.000
Avoiding intimacy	204(46.3)	192(48.9)	181(45.8)	58(27.89	23(45.1)	30(48.4)	688(44.4)	27.9; 5; 0.000
Increase in sexual desire	139(31.5)	139(35.4)	100(25.3)	38(18.2)	12(23.5)	23(37.1)	451(29.1)	26.3; 5; 0.000
Decrease in sexual desire	179(40.6)	192(48.9)	146(37.0)	52(24.9)	23(45.1)	24(38.7)	616(39.7)	34.9; 5; 0.000
Itching in genital region	189(42.9)	178(45.3)	150(38.0)	68(32.5)	28(54.9)	29(46.8)	642(41.4)	16.1; 5; 0.007
Poor memory or forgetfulness	272(61.7)	293(74.6)	323(81.8)	154(73.7)	40(78.4)	48(77.4)	1130(72.9)	45.8; 5; 0.000
Anxious or nervous	295(66.9)	323(82.2)	339(85.8)	174(83.3)	41(80.4)	50(80.6)	1222(78.8)	54.4; 5; 0.000
More impatient	331(75.1)	329(83.7)	341(86.3)	189(90.4)	45(88.2)	47(75.8)	1282(82.7)	33.7; 5; 0.000
Wanting to be alone	333(75.5)	313(79.6)	353(89.4)	192(91.9)	45(88.2)	56(90.3)	1292(83.3)	47.6; 5; 0.000
Headache	380(86.2)	360(91.6)	374(94.7)	202(96.7)	51(100.0)	56(90.3)	1423(91.7)	34.1; 5; 0.000
Low backache or muscle pain	392(88.9)	376(95.7)	389(98.5)	205(98.1)	51(100.0)	61(98.4)	1474(95.0)	53.9; 5; 0.000
Dry skin	369(83.7)	343(87.3)	368(93.2)	176(84.2)	49(96.1)	53(85.5)	1358(87.6)	23.3; 5; 0.000
Patches of darker or lighter skin	331(75.1)	323(82.2)	355(89.9)	178(85.2)	48(94.1)	54(87.1)	1289(83.1)	39.2; 5; 0.000
Increase in flatulence	362(82.1)	329(83.7)	367(92.9)	195(93.3)	46(90.2)	50(80.6)	1349(87.0)	35.3; 5; 0.000
Dyspepsia	371(84.1)	323(82.2)	375(94.9)	198(94.7)	45(88.2)	57(91.9)	1369(88.3)	47.5; 5; 0.000
Feeling tired	393(89.1)	332(84.5)	381(96.5)	202(96.7)	47(92.2)	57(91.9)	1412(91.0)	45.1; 5; 0.000
Lack of energy	384(87.1)	314(79.9)	357(90.3)	189(90.4)	44(86.3)	49(79.0)	1337(86.1)	25.1; 5; 0.000
Weight gain	334(75.7)	253(64.4)	328(83.0)	187(89.5)	39(76.5)	45(72.6)	1186(76.5)	61.7; 5; 0.000
Constipation/Diarrhoea	270(61.2)	219(55.7)	276(69.9)	148(70.8)	28(54.9)	42(67.7)	983(63.4)	25.1; 5; 0.000
Inclination to a fracture in any bone	199(45.1)	184(46.8)	163(41.3)	85(40.7)	28(54.9)	32(51.6)	691(44.6)	7.3; 5; 0.196
Persistent cough	242(54.9)	161(41.0)	126(31.9)	48(23.0)	21(41.2)	34(54.8)	632(40.7)	81.7; 5; 0.000
Sore throat	249(56.5)	153(38.9)	128(32.4)	44(21.1)	19(37.3)	26(41.9)	619(39.9)	91.1; 5; 0.000
Shortness of breath	211(47.8)	133(33.8)	117(29.6)	45(21.5)	19(37.3)	21(33.9)	546(35.2)	53.9; 5; 0.000

"Excessive sweats, 83.8", "poor memory or forgetfulness, 81.8%", "anxiety or nervousness, 85.8%", and dyspepsia, 94.9% were highest in the age group 50-54 when compared to the other age groups, whereas "night sweats, 93.3%", "pins and needles in arms and legs, 88.5%", "heart palpitation, 78.9%", "dizzy spells, 80.9%", "urinating more frequently, 81.8%", "more impatient, 90.4%", "wanting to be alone, 91.9%", "increase in flatulence, 93.3%", "feeling tired, 96.7%", "lack of energy, 90.4%", "weight gain, 89.5%", and "constipation/diarrhoea, 70.8%" were the highest in the age group 55-59 when compared to the other age groups.

"Hot flushes, 100.0%",**"**burning while urinating, 84.3%", "urinary tract infection, 86.3%", "itching in genital region, 54.9%", "headache, 100.0%", "low backache or muscle pain, 100.0%", "dry skin, 96.1%", "patches of darker or lighter skin, 94.1%", and "inclination to a fracture in any bone, 54.9%" were the highest in the age group 60-64 when compared to the other age groups. Further, "an increase in sexual desire", 37.1%, was the highest in the age group 65-69 when compared to the other age groups.

Only 3.5% (n = 54/1551) of the women reported experiencing all of the complaints at a proportion of 100.0%. That is to say, 1497 women (96.5%) had at least one or more complaints. There were no women with the complaints between 1 and 8 (0.0%). Most of the women declared having complaints between 9-14, 15-24 and 25-34 were in the older age group 50-64 when compared to the younger age group 40-49 (60% and over) (p < 0.05). There were no women with 0.0% complaints (Unshown data in Table).

The incidence of climacteric complaints and the percentage of women experiencing each symptom are given in Table 5. Among Turkish women, hot flushes was the most common complaint occurring in 96.5% of women (32.9% to a severe degree, 43.1% to a moderate degree, and 20.4% to a mild degree). This complaint was followed by low backache or muscle pain 95.0% (25.9% severe, 46.0% moderate and mild 23.1%), headaches 91.7% (21.9% severe, 34.9% moderate and 34.9% mild), and feeling tired 91.0% (15.3% severe, 38.6% moderate and 37.1% mild).

**Table 5:** Menopausal symptoms that women aged 40 and over experienced

Menopausal symptoms (Alpha coefficient: 0.931)	Severe n(%)	Moderate N(%)	Mild n(%)	None n(%)	Sum of complaints n(%)	mean	SD
Hot flushes	511(32.9)	669(43.1)	317(20.4)	54(3.5)	1497(96.5)	2.0554	.8188
Night sweats	446(28.8)	533(34.4)	394(25.4)	178(11.5)	1373(89.5)	1.8040	.9812
Excessive sweats	410(26.4)	510(32.9)	296(19.1)	335(21.6)	1216(78.4)	1.6415	1.0915
Sleep disturbances	370(23.9)	520(33.5)	418(27.0)	243(15.7)	1308(84.3)	1.6557	1.0084
Pins and needles in arms and legs	318(20.5)	491(31.7)	477(30.8)	265(17.1)	1286(82.9)	1.5558	.9997
Palpitations	241(15.5)	380(24.5)	498(32.1)	432(27.9)	1119(72.1)	1.2772	1.0339
Loss of appetite	54(3.5)	195(12.6)	460(29.7)	842(54.3)	709(45.7)	.6525	.8292
Dizzy spells	94(6.1)	352(22.7)	716(46.2)	389(25.1)	1162(74.9)	1.0974	.8433
Vaginal dryness	70(4.5)	244(15.7)	525(33.8)	712(45.9)	839(54.1)	.7885	.8676
Burning while urinating	136(8.8)	407(26.2)	591(38.1)	417(26.9)	1134(73.1)	1.1689	.9241
Urinating more frequently	168(10.8)	492(31.7)	477(30.8)	414(26.7)	1137(73.3)	1.2669	.9730
Urinary tract infection	149(9.6)	440(28.4)	501(32.3)	461(29.7)	1090(70.3)	1.1786	.9664
Avoiding intimacy	72(4.6)	208(13.4)	408(26.3)	863(55.6)	688(44.4)	.6705	.8764
Increase in sexual desire	51(3.3)	105(6.8)	295(19.0)	1100(70.9)	451(29.1)	.4242	.7598
Decrease in sexual desire	85(5.5)	130(8.4)	400(25.8)	936(60.3)	615(39.7)	.5899	.8596
Itching in genital region	71(4.6)	163(10.5)	408(26.3)	909(58.6)	642(41.4)	.6106	.8503
Poor memory or forgetfulness	192(12.4)	455(29.3)	483(31.1)	421(27.1)	1130(72.9)	1.2695	.9940
Anxious or nervous	212(13.7)	489(31.5)	521(33.6)	329(21.2)	1222(78.2)	1.3765	.9659
More impatient	292(18.8)	461(29.7)	529(34.1)	269(17.3)	1282(82.7)	1.5003	.9869
Wanting to be alone	298(19.2)	468(30.2)	526(33.9)	259(16.7)	1292(83.3)	1.5190	.9841
Headache	340(21.9)	542(34.9)	541(34.9)	128(8.3)	1423(91.7)	1.7054	.9010
Low backache or muscle pain	401(25.9)	714(46.0)	359(23.1)	77(5.0)	1474(95.0)	1.9278	.8269
Dry skin	178(11.5)	426(27.5)	754(48.6)	193(12.4)	1358(87.6)	1.3798	.8452
Patches of darker or lighter skin	126(8.1)	425(27.4)	738(47.6)	262(16.9)	1289(83.1)	1.2676	.8347
Increase in flatulence	150(9.7)	579(37.3)	620(40.0)	202(13.0)	1349(87.0)	1.4365	.8369
Dyspepsia	199(12.8)	598(38.6)	572(36.9)	182(11.7)	1369(88.3)	1.5248	.8609
Feeling tired	238(15.3)	598(38.6)	576(37.1)	139(9.0)	1312(91.0)	1.6028	.8521
Lack of energy	225(14.5)	557(35.9)	555(35.8)	214(13.8)	1337(86.2)	1.5113	.9036
Weight gain	198(12.8)	393(25.3)	595(38.4)	365(23.5)	1186(76.5)	1.2734	.9619
Constipation/Diarrhoea	119(7.7)	289(18.6)	575(37.1)	568(36.6)	983(63.4)	.9736	.9270
Inclination to a fracture in any bone	61(3.9)	192(12.4)	438(28.2)	860(55.4)	691(44.6)	.6480	.8439
Persistent cough	41(2.6)	175(11.3)	416(26.8)	919(59.3)	632(40.7)	.5732	.7933
Sore throat	27(1.7)	176(11.3)	416(26.8)	932(60.1)	619(39.9)	.5474	.7613
Shortness of breath	24(1.5)	160(10.3)	362(23.3)	1005(64.8)	546(35.2)	.4861	.7412

Analysis of the study also revealed statistically significant relationships between almost all the symptoms and the women's menopause status, apart from the symptoms 'lack of energy' and 'constipation/diarrhoea'. Comparing women in the postmenopausal-surgical group with those in the other groups (women in premenopausal, premenopausal, and postmenopausal-natural), we found that women in this group had the highest proportion of complaints for all categories except for just 3 items, 'persistent cough', 'sore throat' and 'shortness of breath'. This was in contrast to women in premenopause who reported these complaints in the highest proportions. Detailed data are presented in Table 6.

**Table 6:** The relationships between menopausal symptoms and menopause status

Symptoms	Premenopause n(%) 272(17.5)	Perimenopause n(%) 275(17.7)	Postmenopause-natural n(%) 823(53.1)	Postmenopause-surgical n(%) 182(11.7)	**Statistical analysis x^2^; DF; p**
Hot flushes	259(95.6)	260(94.5)	797(96.8)	181(99.5)	8.8; 3; 0.032
Night sweats	248(91.5)	237(86.2)	709(86.1)	179(98.4)	25.7; 3; 0.000
Excessive sweats	220(81.2)	212(77.1)	608(73.9)	176(96.7)	47.5;3;0.000
Sleep disturbances	239(88.2)	241(87.6)	649(78.9)	179(98.4)	51.2;3;0.000
Pins and needles in arms and legs	239(88.2)	230(83.6)	642(78.0)	175(96.2)	41.9;3;0.000
Hearth beating	196(72.3)	199(72.4)	565(68.7)	159(87.4)	25.9;3;0.000
Loss of appetite	129(47.6)	143(52.0)	327(39.7)	110/60.4)	32.5;3;0.000
Dizzy spells	186(68.6)	209(76.0)	607(73.8)	160(87.9)	22.8;3;0.000
Vaginal dryness	161(59.4)	151(54.9)	409(49.7)	118(64.8)	18.1;3;0.000
Burning while urinating	175(64.6)	166(60.4)	638(77.5)	155(85.2)	54.4;3;0.000
Urinating more frequently	180(66.4)	179(65.1)	615(74.7)	163(89.6)	41.5;3;0.000
Urinary tract infection	169(62.4)	166(60.4)	601(73.0)	154(84.6)	41.9;3;0.000
Avoiding intimacy	119(43.9)	132(48.9)	340(41.3)	97(53.3)	10.5;3;0.015
Increase in sexual desire	81(29.9)	89(32.4)	202(24.5)	79(43.4)	27.9;3;0.000
Decrease in sexual desire	105(38.7)	118(42.9)	295(35.8)	97(53.3)	20.5;3;0.000
Itching in genital region	118(43.5)	109(39.6)	304(36.9)	111(61.0)	36.4;3;0.000
Poor memory or forgetfulness	167(61.6)	192(69.8)	614(74.6)	157(86.3)	36.4;3;0.000
Anxious or nervous	183(67.5)	209(76.0)	661(80.3)	169(92.9)	44.5;3;0.000
More impatient	202(74.5)	224(81.5)	690(83.8)	166(91.2)	22.8;3;0.000
Wanting to be alone	206(76.0)	207(75.3)	708(86.0)	171(94.0)	42.3;3;0.000
Headache	237(87.5)	245(89.1)	770(93.6)	171(94.0)	13.9;3;0.003
Low backache or muscle pain	238(87.8)	253(92.0)	803(97.6)	180(98.9)	52.2;3;0.000
Dry skin	225(83.0)	228(82.9)	737(89.6)	168(92.3)	17.3;3;0.001
Patches of darker or lighter skin	194(71.6)	218(79.3)	708(86.0)	169(92.9)	45.8;3;0.000
Increase in flatulence	222(81.9)	227(82.5)	731(88.8)	169(92.9)	18.3;3;0.000
Dyspepsia	225(83.0)	237(86.2)	738(89.7)	169(92.9)	13.6;3;0.003
Feeling tired	241(88.9)	238(86.5)	763(92.7)	170(93.4)	12.4;3;0.006
Lack of energy	233(86.0)	239(86.9)	703(85.4)	162(89.0)	1.76;3;0.624
Weight gain	206(76.0)	202(73.5)	623(75.7)	155(85.2)	9.4,4;0.025
Constipation/Diarrhoea	169(62.4)	174(63.3)	515(62.6)	125(63.4)	2.6;3;0.465
Inclination to fracture in any bone	123(45.4)	132(48.0)	331(40.2)	105(57.7)	20.4;3;0.000
Persistent cough	156(57.6)	134(48.7)	254(30.9)	88(48.4)	76.7;3;0.000
Sore throat	166(61.3)	132(48.0)	239(29.0)	82(45.1)	101.5;3;0.000
Shortness of breath	132(48.7)	119(43.3)	217(26.4)	78(42.9)	62.4;3;0.000

Over two third of the women reported that their social life (69.5%) was not affected after they had gone through the menopause. About one third of those who reported that their social life was affected after experiencing the menopause (30.5%) were in the youngest age group 40-44 (32.8%), followed by the age group 45-49 (27.7). The age group 60-64 reported being affected the least (3.0%) (p < 0.001). About 40% of the women (36.9%) indicated that their level of coping with menopause symptoms was good, moderate 65.2%, and low 14.3%. Most of those whose coping level was good were in the age group 40-54, in particular the age group 40-44. A lot of women (82.9%) indicated being affected by menopause or menopause complaints to a severe or moderate level, and 14.3% to a mild degree. About one third of those who had been distressed by menopause or menopausal complaints to a severe degree were in the age group 55-59, followed by the age group 50-54 (32.4% and 31.6%, respectively). A large section of the women (n = 1438, 92.7%) reported having received medical or psychological help from the following persons due to menopause or menenopause complaints: gynecologist (79.1%), general practitioner (5.7%), nurse (2.9%), family practitioner (2.5%), store selling dietary supplements (1.9%), psychologist (1.8), urologist (1.8), Psychiatrist (1.3%), pharmacist (1.3%), Muslim preacher (0.9%), and midwife (0.8%). Approximately 50% of those in the age group 40-44 had sought medical or psychological help (48.7%), followed by the age group 45-49 (18.6%) (Unshown data in Table).

The majority of the respondents indicated that their level of information about menopause was average (52.1%), followed by low (39.3%) and high (8.6%). Most respondents reported that mass media (television, 71.2%; newspapers, 37.4%; magazines, 20.2%; radio, 12.8%; internet, 4.2%) was the major source of their information about menopause (58.8%), followed by friends/relatives and general practitioners (34.9% and 27.9%, respectively), gynecologists (21.5%), and nurses (15.2%). Upon being asked with whom the women had discussed the climacteric, most had not discussed it with anybody (44.3%), 52.5% had discussed it with their relatives, followed by friends (43.8%), and husband or partner (28.5%). Most indicated that they desired to learn more (91.5%).

## Discussion and Conclusions

This paper, the first large scale Turkish epidemiological study of this region, reports data from a population-based study on menopause symptoms, features, and both people's attitudes and misconceptions related to menopause among the general population in a city of western Turkey. The participation rate was high (78.1%) and detailed information on a variety of menopausal symptoms was obtained. The results demonstrate the women's willingness to discuss menopause or menopausal symptoms.

Culture and ethnicity are reflected in beliefs, traditions, language, and social structure. In some cultures, the loss of regular bleeding is connected with a communal benefit since menopause means the end of "the days of uncleanness", while in more procreation-minded societies, menopause is seen mainly in a negative way because it means the end of fertility and the end of youth [24]. This finding was compatible with our study findings indicating that 90.7% of the women surveyed see menopause as "the end of youth"; 85.8% see it as "the beginning of getting older", and 97.6% as "the end of fecundity". On the other hand, the prevalence of those who reported menopause as "the end of uncleanness" was 96.4% which could be a cultural influence indicating that the loss of regular bleeding is related to a social gain. It also shows that the Turkish women interviewed in our study see the regular bleeding as unclean in line with the study showing that 83.42% women were happy about the cessation of menses and they did not wish to have menses again [28].

In numerous studies, many women held the opinion that the climacteric is a normal phase in a woman's life (75.7%), and feminity is not lost in the climacteric period (78.8%) [21,29]. This is in line with our study findings. In addition, the proportions of those agreeing to the items 'a woman feels herself to be in the prime of life' and 'It is a period that must be experienced' were rather high (89.4% and 86.4%, respectively). This result shows that Turkish women see menopause as the time of growing into a wise woman, and that Turkish women assume the wise woman approach, in line with a qualitative study of Jordanian women's perceptions [30].

However, the proportion of those who reported "the end of fecundity" was rather high (97.6%), which was not compatible with our results. In our study, younger age groups had more negative opinions of the climacteric with regards to feminity, consistent with an Estonian study [21]. It may be hypothesized that ethnic and cultural factors affect women's attitudes to acceptance of the menopause, menopausal symptoms, and their compliance and expectations.

In this study, about one third of the women (32.0%) believed that "the rate of divorce could increase in this period", and also that the climacteric is a "period for cuckolding" (21.3%). These results reveal that some menopausal women see menopause as the end of sexuality.

Although the women in this study live in a Muslim country and they ware also Muslims, only 22.5% reported that they believed in the relationship between God and the climacteric, as believed in common misconceptions. In other words, about 70% of the women disagreed with there being a connection between God and the climacteric.

On the other hand, most women said that cessation of menstrual periods was the most positive thing because they do not have to wait for monthly bleeding, use sanitary equipment, take birth control methods, and they are not at risk for getting pregnant. These results also show that women in the menopausal period perceive menopause as a heavy burden and a problem that must be finished as quickly as possible.

In the current study, the most frequently reported menopausal symptoms were "hot flushes", "headache", "low backache or muscle pain" and 'feeling tired". These results are in line with many reports indicating that these symptoms, particularly hot flushes, are the most common symptoms [30]. In parallel, in a study concerning climacteric symptoms in Turkish women [15], the most common symptoms were muscle and- or joint and- or bone pain (82.3%) and hot flushes (73.9%). In another survey, various different problems were reported by 86% of the women, mainly hot flushes [17]. Moreover, these results are compatible with some studies reporting that lumbago or low back pain is the most frequent menopausal symptom in Taiwan, and hand joints in Korea [31]. In contrast, some researchers have indicated that hot flushes were indicated by only 45% of subjects, with the loss of libido as the main complaint for Movima climacteric women in Bolivia [24]. These results were not compatible with our study findings in which we found that hot flushes were the main symptom and loss of libido was an uncertain symptom with 39.7%. In our study, 45.9% (839/1551) women reported no sexual symptoms and 54.1% reported mild to severe sexual symptoms, ratios which were not in line with a study indicating that 30% women reported no vaginal dryness and 25% reported moderate to extremely bothersome sexual symptoms. Many Asian studies reported rather low prevalences of 9.8%-38.5% for hot flushes. These contradictory results may be explained by our uncertainty concerning precisely which conditions change the incidence of vasomotor symptoms; cultural, social, economic, psychic or physiological differences [32]. As a result, a great deal of the variance in reports of menopausal symptom prevalence, type, and severity may be due to study design, including whether the study is cross-sectional or longitudinal, and whether the study population is a community-based or clinical sample.

Previous studies investigating factors influencing the prevalence, type, and severity of menopausal symptoms have found that lower educational level, lack of employment outside the home, and lower socioeconomic status are associated with increased prevalence and severity of menopausal symptoms [10,33,34]. Polit and LaRocco (1980) investigated the impact of demographic and personality variables on the nature and intensity of subjectively perceived menopausal symptoms. They found that women who reported a higher number of menopausal symptoms tended to be less educated, that they were less likely to be working, and that thay viewed themselves in poorer health than women with fewer symptoms [35]. Our findings are consistent with the prior studies.

One study that addressed the question of health care utilization found that in addition to menopausal symptoms, being a homemaker, and having lower self-assessed health was associated with problem-related health care utilization among women [36]. Another utilization study found that women seeking assistance from a health institution or health personnel tended to be married, have children, and be relatively well educated [37]. Similarly, our study showed that most of those utilizating health care were postmenopausal-natural and surgical women (94.4% and 98.9%, respectively) when compared to premenopausal and perimenopausal women (93.8% and 82.3%, respectively) (x2 = 57.9; DF = 3; p = 0.000). Most of those utilizating health care were married when compared to single (93.1% and 90.2%, respectively) although the comparison was not significant (x2 = 2.1; DF = 1; p = 0.144). Women who were parous tended utilizating health care more than nonparous women (93.4% and 70.8 (respectively) (x^2^= 35.1; DF = 1; p = 0.000). When compared to illiterate women (79.7%), the whole primary, high school and university educated women (94.0%, 91.0% and 92.8%, respectively) were found to utilize more health care. In addition, our results are compatible with the study by Wells et al (1989) where they found that those who were less acculturated had significantly lower probabilities for health care utilization [38].

Changes in the age of the natural menopause may be due to social, economic, environmental or genetic factors [24]. In this study, the mean natural menopausal age was 52.9, which is similar to those of Western women with a mean age of 51.4 [39]. This age was 47 for South-American women, and 50 for Swedish women [40]. A study in seven South-East Asian countries found that the earliest age at menopause was observed in the Philippines (47-48 years) and the very last in Taiwan (52 years) [14]. One possible explanation for these large changes in menopausal ages may be related to the fact that ethnic, biological and cultural background may have an impact on age at menopause [24,41]. This interesting aspect, in our opinion, merits further investigation.

In the current study, the proportion of hormone therapy use was only 35.4%, consistent with the study of Lewin et al. (2003) [42]. These similar results merit further investigation into what shapes attitudes towards hormone therapy. In the present study, hormone use was higher in postmenopausal-natural women (44.4%) than in pre- and perimenopausal women (20.7% and 23.8%, respectively). furthermore, vasomotor symptoms such as hot flushes, night sweats and excessive sweats were significantly lower for postmenopausal-natural women taking hormone therapy than those who had not. This may explain the lower prevalence of vasomotor symptoms in this population. Such an association could be partially mediated through the effect of stress on catecholamine and oestrogen changes [5].

In working or well paid employed women, the prevalence of symptoms was less reported. Perhaps a more active lifestyle, with a focus on work may distract women from noticing some of the adverse experiences. Being unemployed for either personal or health reasons was also a source of stress [5].

In this study, more than half of the women reported that they had not used any hormone therapy (64.5%), any medicine for the distress that menopause causes (83.9%), any medicine for the insomnia that menopause causes (72.1%), and any calcium supplement concerning menopause (47.9%). These results are compatible with a study conducted in Isra [28], indicating that 75.20% of women were not taking any medicine for symptoms. This indicates that women are not willing to take medication.

These results may be of particular relevance for all the social, health, governmental, and municipal managers, but in particular health workers working in primary health care. Given the facts that the cost of health care services has become a heavy burden for societies, clinicians have less time to spend on a patient, and increasing numbers of patients visit primary health care centers with vague symptoms, a fast, cost-effective first screening for menopause would be a welcome aid [43,44]. Further studies on the impact of hormonal changes, diet, lifestyle and sociocultural characteristics are also necessary to further understand mid-life experiences.

For such a study, which was conducted in only one city, it is difficult to generalize about the climacteric among Turkish women. However, the major interest of this survey is the opportunity to offer more information on Turkish women's attitude to menopause.

Such a study may suffer from methodological shortcomings. The most important is that data is self-reported in a retrospective way, and therefore, may tend to underestimate the real values about principally attitudes and misconceptions, and this illustrates that there are many that don't actually recognize their own menopause.

Although the data obtained was based on subjective experiences, self-reporting is the most practical way to obtain information on symptom reporting in large-scale population-based studies. The structured questionnaire and symptom list facilitated confidence in our having obtained information in a standardized manner.

## Competing interests

The authors declare that they have no competing interests.

## Authors' contributions

AU conceived of the study, OO and AU participated in its design and coordination, sequence alignment, collected the data and drafted the manuscript, OO participated in its coordination and collected the data, AU participated in the design of the study, performed the statistical analyses and collected the data, EDF and AG participated in its coordination and collected the data. All authors read and approved the final manuscript.

## Pre-publication history

The pre-publication history for this paper can be accessed here:

http://www.biomedcentral.com/1472-6874/10/1/prepub

## Supplementary Material

Additional file 1 The questionnaires used to assess menopausal symptoms in Turkish women. Click here for file
