# Supplementary material for: Menopause status and attitudes in a Turkish midlife female population: an epidemiological study
Source: BMC Womens Health. 2010 Jan 11;10:1. doi: 10.1186/1472-6874-10-1 (PMC2822813; doi:10.1186/1472-6874-10-1)
Supplement: Additional file 1 — The questionnaires used to assess menopausal symptoms in Turkish women. [file 1472-6874-10-1-S1.DOC]

**THE QUESTIONNAIRES**

****Your attitudes towards menopause****

|  | I disagree | I neither agree  nor disagree | I agree |
| --- | --- | --- | --- |
| The end of youth |  |  |  |
| The end of life |  |  |  |
| The beginning of getting older |  |  |  |
| The end of fecundity |  |  |  |
| The d[**ivorce rate**](http://www.tureng.com/search/divorce+rate) could increase in this period |  |  |  |
| I cannot endure problems from child or spouse in this period |  |  |  |
| The end of uncleanness days |  |  |  |
| The beginning of distress |  |  |  |
| The period for cuckolding |  |  |  |
| I will not be obliged to use [**sanitary pad**](http://www.tureng.com/search/sanitary+pad)s |  |  |  |
| I will experience no distress such as irregular menses |  |  |  |
| I will not be in need of using birth control methods |  |  |  |
| I will be at no risk for getting pregnant |  |  |  |
| Woman feels herself to be [**in the prime of life**](http://www.tureng.com/search/in+the+prime+of+life) |  |  |  |
| A period that must be experienced |  |  |  |
| It is not necessary to consult a doctor for treatment in this period |  |  |  |
| Hormones should be given to all menopausal women |  |  |  |
| Hormones should be given to all women having menopausal symptoms |  |  |  |
| It is the punishment that God gave women |  |  |  |
| The end of unclean days is a reward that God gave to women |  |  |  |

**Menopausal symptoms that you experienced**

|  | Severe | Moderate | Mild | None |
| --- | --- | --- | --- | --- |
| Hot flushes |  |  |  |  |
| Night sweats |  |  |  |  |
| Excessive sweats |  |  |  |  |
| Sleep disturbances |  |  |  |  |
| [**Pins and needles**](http://www.tureng.com/search/pins+and+needles) in arms and legs |  |  |  |  |
| Palpitations |  |  |  |  |
| Loss of [**appetite**](http://www.tureng.com/search/appetite) |  |  |  |  |
| Dizzy spells |  |  |  |  |
| Vaginal dryness |  |  |  |  |
| Burning while urinating |  |  |  |  |
| Urinating more frequently |  |  |  |  |
| Urinary tract infection |  |  |  |  |
| Avoiding intimacy |  |  |  |  |
| Increase in sexual desire |  |  |  |  |
| Decrease in sexual desire |  |  |  |  |
| Itching in genital region |  |  |  |  |
| Poor memory or forgetfulness |  |  |  |  |
| Anxious or nervous |  |  |  |  |
| More impatient |  |  |  |  |
| Wanting to be alone |  |  |  |  |
| Headache |  |  |  |  |
| Low backache or muscle pain |  |  |  |  |
| Dry skin |  |  |  |  |
| Patches of darker or lighter skin |  |  |  |  |
| Increase in flatulence |  |  |  |  |
| [**Dyspepsia**](http://www.tureng.com/search/dyspepsy) |  |  |  |  |
| Feeling tired |  |  |  |  |
| Lack of energy |  |  |  |  |
| Weight gain |  |  |  |  |
| Constipation/Diarrhoea |  |  |  |  |
| Inclination to a fracture in any bone |  |  |  |  |
| Persistent cough |  |  |  |  |
| Sore throat |  |  |  |  |
| Shortness of breath |  |  |  |  |
